# The lethal sex gap: COVID-19

**DOI:** 10.1186/s12979-020-00183-z

**Published:** 2020-05-21

**Authors:** Eladio J. Márquez, Jennifer Trowbridge, George A. Kuchel, Jacques Banchereau, Duygu Ucar

**Affiliations:** 1 Sanofi US, Cambridge, MA 02139 USA; 2 The Jackson Laboratory, Bar Harbor, ME 04609 USA; 3 University of Connecticut Center on Aging, UConn Health Center, Farmington, CT 06030 USA; 4 The Jackson Laboratory for Genomic Medicine, Farmington, CT 06030 USA

**Keywords:** Immunosenescence, Inflammaging, COVID-19, Sex differences

## Abstract

While Coronavirus disease 2019 (COVID-19), caused by severe acute respiratory syndrome coronavirus 2 (SARS-CoV-2), is disrupting lives across the globe for everyone, it has a more devastating impact on the health of older adults, especially that of older men. This pandemic has highlighted the crucial importance of considering an individual’s age and biological sex in the clinic in addition to other confounding diseases (Kuchel, G.A, J Am Geriatr Soc, 67, 203, 2019, Tannenbaum, C., Nature, 575 451-458, 2009) As an interdisciplinary team of scientists in immunology, hematology, genomics, bioinformatics, and geriatrics, we have been studying how age and sex shape the human immune system. Herein we reflect on how our recent findings on the alterations of the immune system in aging might contribute to our current understanding of COVID-19 infection rate and disease risk.

## Immune system aging and COVID-19

Many parameters likely contribute to the etiology of the COVID-19 disease. The number of viral particles (i.e., viral load) and the mode of infection might explain why health care workers are at a higher risk; differences in the genome of the virus strains or the genome of the host (i.e., genetic makeup of the patients) might account for some of the variation observed across countries and populations. At the individual level, in addition to the aforementioned factors, a person’s immune system status is also an important predictor of the disease outcome, which can be shaped by the individual’s age, sex, as well as the existence of co-morbidities.

COVID-19 shows differences in terms of which populations are vulnerable when compared to previous pandemics. Pregnant women were at increased risk during the H1N1 pandemic in 2009 [1], whereas the H1N1 pandemic of 1918 (known as Spanish flu) particularly affected younger individuals: 15- to 34-year-olds [2]. While COVID-19 appears to have a milder effect on these populations so far [3], increasing age of an individual clearly stands out as an important predictor of vulnerability for COVID-19. According to data from China, the COVID-19 death rate is 3.6% for individuals in their 60s, 8% for those in their 70s, 15% for individuals older than 80, yet ~ 0.5% for individuals in their 40s (https://www.worldometers.info/coronavirus/coronavirus-age-sex-demographics/). Such an age discrepancy is also found in other countries. Most notably in Italy, case fatality rate for individuals in their 70s and 80s was reported as 25% and 31% respectively (https://www.epicentro.iss.it/coronavirus/) and the average age of patients dying from COVID-19 was 79 (based on 19,996 deaths on April 16th). Many factors can accelerate an individual’s biological age, including diet, exercise, lifestyle choices (smoking) and co-morbidities (diabetes, obesity) [4]. Therefore, increasing age (biological and chronological) likely predispose individuals to severe COVID-19 outcomes.

Our immune system is composed of two distinct arms with different functions: adaptive and innate immunity. Our team [5] and other investigators [6–13] have documented that, with aging, both arms of our immune systems go through changes in cellular composition and in function. Innate immunity is the first line of defense against dangerous invaders such as SARS-CoV-2, the virus that causes COVID-19. The innate immune system acts through its ability to capture and inactivate pathogens and to launch inflammation. Typically, inflammatory responses are acute; they last for a short time and lead to a rapid accumulation of immune cells and proteins at the site of injury to remove the invader and start the healing process. When acute responses are insufficient, inflammatory responses can prolong thereby affecting numerous cellular components. Aging has been linked with such chronic activation of innate immunity, associated with low-grade and systemic (body-wide) increases in inflammation (coined “inflamm-aging”) that can be detrimental for the body [8, 14, 15] and also conserved across tissues and organisms [16]. In other words, a biological response that is beneficial in a healthy immune system in youth can become a potential liability in older age [17].

Adaptive immune cells are mobilized when the innate immune system is insufficient to defeat a threat. Whereas innate immunity acts quickly to recognize microbes by their general features, adaptive immune cells, B and T cells, can eliminate a threat with precision by specifically recognizing foreign substances associated with a certain threat (for example, a short protein fragment - an antigen - unique to SARS-CoV-2). After successfully clearing a threat/infection, the body maintains a reserve of “memory cells” which remember how to recognize the threat and clear it quickly in case of future invasions. The delicate co-operation and balance between innate and adaptive immune cells are critical to orchestrate an effective immune response at any age. With increasing age, this balance is disrupted. In addition to chronic activation of innate immunity, adaptive immune functions decline with age [8, 18, 19]. These declines affect the adaptive immune system’s ability i) to recognize novel threats due to a loss in the number of cells that can be educated to recognize novel threats (i.e. naïve cells), and ii) to mount strong responses due to the accumulation of over-stimulated and dysfunctional, “exhausted” immune cells. Recent data suggest that with aging, some adaptive cells also change their functionality and gain innate-like functions [20]. Preliminary data from COVID-19 patients in Wuhan suggest that disease severity was associated with reduced numbers of T cells (naïve and memory helper T cells, regulatory T cells) in the blood (*n* = 44; 27 severe, 17 non-severe) [21]. In alignment with the global patterns, severe cases were older than non-severe cases (median age 61 versus 53) and included more men than women (54% men) (*n* = 452; 286 severe, 166 non-severe) [21].

**Fig. 1:**
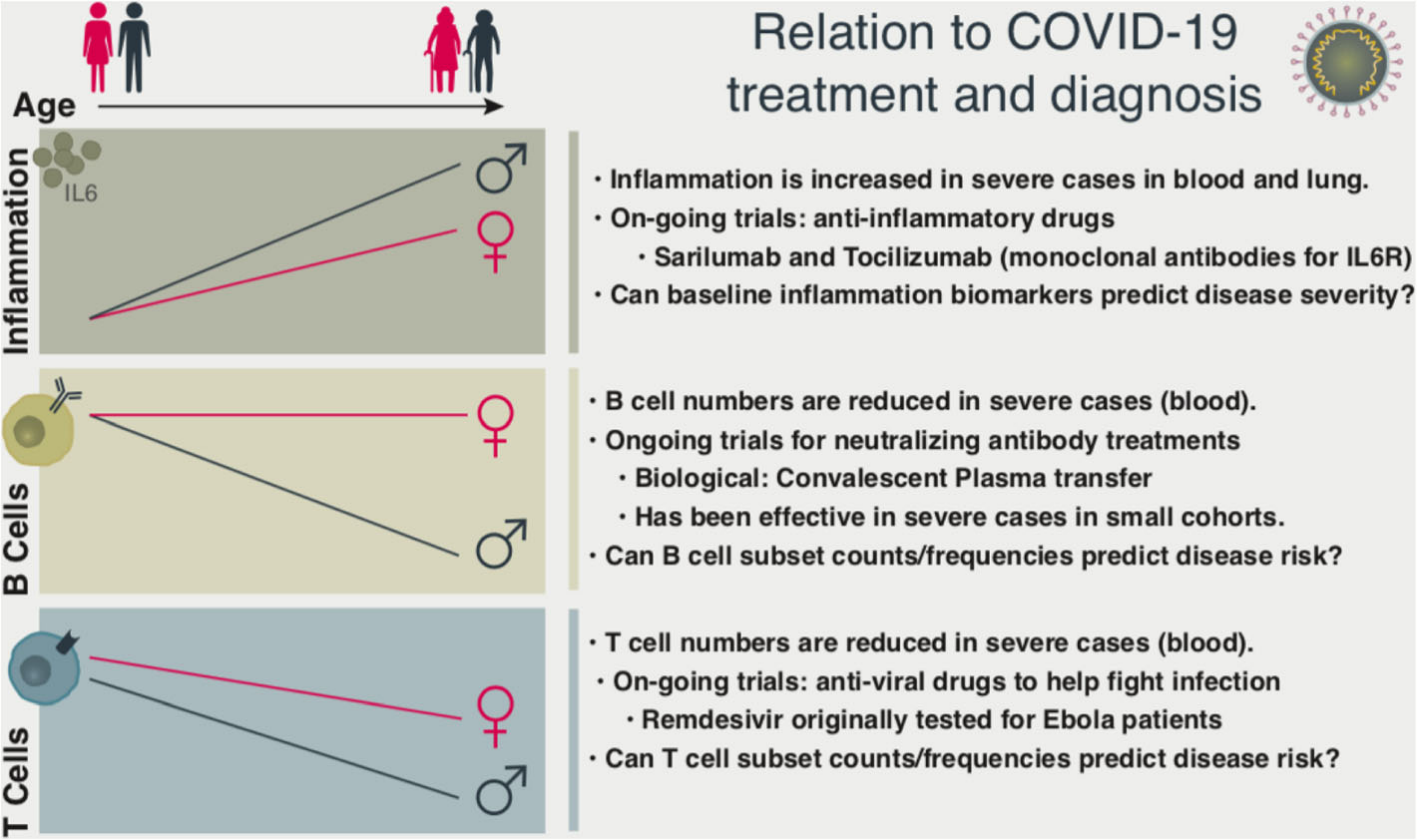
Summary of age- and sex-related changes in the immune system in relation to COVID-19. Inflammation increases with age in both sexes with age, albeit at higher rates in men. Severe COVID-19 cases have been associated with increased inflammation markers in blood and lung. The efficacy of anti-inflammatory drugs are in trials for COVID-19 patients. B cell numbers decline with age specifically in men. Severe cases of COVID-19 have been associated with reduced B cell numbers. Convalescent plasma transfer is being investigated as a potential therapy for COVID-19 patients. T cell functions decline with age, including declining naïve T cell numbers as well as reduced ability to mount strong responses to immune threats. Declines in T cell numbers have been observed in severe COVID-19 patients. Anti-viral drugs can be useful to boost immune cell responses. These observations might also be useful in assessing disease risk in healthy individuals by studying their immune status. For example, individuals who have less B/T cells, more inflammation or more monoclonal hematopoiesis, might be more prone to devastating health consequences of COVID-19

Older age has been associated to increased risk for developing acute respiratory distress syndrome (ARDS) [22] (*n* = 201; 84 with ARDS) in COVID-19 patients, which are the most severe cases that require ICU admission and oxygen therapy [23] (*n* = 41; 13 requiring ICU). Severe cases have also been associated with increased levels of blood pro-inflammatory cytokines [21–24]. Two of the pro-inflammatory proteins that were elevated in the blood of severe cases [21] have been associated with low physical performance among older adults: IL-6, C-reactive protein (CRP) [15] and are considered as biomarkers of inflamm-aging. Serum concentrations of these molecules have also been linked to obesity and visceral adiposity [25, 26]. Furthermore, systemic inflammation has been proposed as a risk factor for multiple diseases [14]. Further research is needed to establish whether the baseline levels of these proteins in the blood can predict disease severity for COVID-19. Clinical trials are ongoing to assess the efficacy of reducing systemic inflammation using anti-inflammatory drugs for severe COVID-19 patients, including humanized monoclonal antibodies that bind and block the interleukin-6 receptor (IL-6R): Tocilizumab and Sarilumab (Fig. 1).

Data from a small cohort suggest that, in severe cases, pro-inflammatory innate immune cells expand in the lung (*n* = 6 patients, 3 of which severe; *n* = 8 healthy controls), whereas T cells expand in milder cases [27]. As expected, patients with severe disease were older than those with milder disease (median age 63 versus 36). This stark difference in the mechanisms of response to the virus between older and young patients is consistent with age-related changes in the immune system: chronic activation of innate immunity and functional declines in adaptive immunity. Together these age-related changes in the immune system deteriorate an individual’s health and might impair responses to SARS-CoV-2 in older adults.

## Sex differences in infectious diseases and COVID-19

In addition to an individual’s age, biological sex is an important determinant of COVID-19 disease severity. In China, across all age groups, the death rate among confirmed cases is 2.8% for women and 4.7% for men (https://www.worldometers.info/coronavirus/coronavirus-age-sex-demographics/). In Italy, although half of confirmed cases are men, men account for 65% of all deaths (as of April 16th) (https://www.epicentro.iss.it/coronavirus/). Even in countries that reported fewer infected men than infected women, such as Spain (49%) and Switzerland (47%), men account for 63 and 62% of deaths, respectively based on mid-April data statistics (http://globalhealth5050.org/covid19/). This pattern is generally consistent around the world, including in the USA (https://www.nytimes.com/2020/04/07/health/coronavirus-new-york-men.html): on average, for each woman killed by COVID-19, 1.5 to 2 men succumb to the virus.

It has long been known that men and women differ in terms of risk and severity for diseases involving the immune system [28]. Women are disproportionately affected by auto-immune disorders, whereas men are more susceptible to infectious diseases, [29], both in terms of their intensity (the severity of the disease in an individual) and prevalence (the number of infected people in a population) [30]. Although epidemiological data for COVID-19 cases are still missing for many countries, among the countries that have a high number of infections and have reported such information, the sex differences are striking (http://globalhealth5050.org/covid19/), in worst cases, the difference is two-fold between men and women in terms of death rate. Why is COVID-19 more dangerous for men, especially for older men?

The reasons for sex differences in COVID-19 are likely multifactorial and include genetics, lifestyle differences, co-morbidities, and hormones [28, 29]. For example, in China, men smoke at much higher rates than women. Smoking rates have been associated with increased activity of ACE2 [31] - the receptor protein that is expressed on the surface of human cells and is used as a “door” by the SARS-CoV-2 virus. Interestingly, this gene is located on the X chromosome. However it is unclear whether the sex- and age-related changes in ACE2 activity is biological [32] or is attributable to confounding factors (e.g., cigarette smoking [31]). Furthermore, although it has been speculated that sex differences in China might stem from differences in cigarette smoking rates between men and women, data on this are inconclusive thus far [33]. Men also have a greater incidence of chronic illnesses such as ischemic heart disease that can impact one’s ability to fight and survive the COVID-19 infection.

## The role of genetics in sex differences

X chromosome contains the largest number of immune-related genes in the whole genome [34], including genes that are involved in innate (e.g., pattern recognition receptors TLR7 and TLR8 that are highly expressed on monocytes) and adaptive immune responses (e.g., chemokine receptor CXCR3 that is highly expressed on effector T cells). It is suggested that X chromosome is partially responsible for the hyper-responsiveness of the female immune system – hence the increased incidences of auto-immune diseases in women – by contributing to the breakdown of self tolerance (i.e., immune system tolerating cells/molecules from self) [35]. Women (XX genotype) carry twice as many X-linked genes (> 1000 genes) compared to men (XY genotype). Expression levels of X-linked genes are balanced between sexes via a process called X chromosome inactivation (XCI), which transcriptionally inactivates one copy of the X chromosome. XCI is established during embryonic development and mostly maintained throughout lifespan [36]. Although XCI is generally uniform across tissues, a recent study conducted from 449 individuals in 29 tissues showed that there are tissue- and individual-specific heterogeneity in XCI patterns [32]. The role of this heterogeneity in human health particularly in COVID-19 is not known yet. Furthermore, some genes – particularly the ones that have Y chromosome homologues – can escape XCI, known as escapees. Hence, these genes can be expressed from two copies: maternal and paternal. Escapee genes might also be contributing to diseases. For example, single-cell analyses of women blood-derived immune cells uncovered that the TLR7 gene is expressed from both maternal and paternal copies in primary B cells, monocytes, and plasmacytoid dendritic cells [37]. Furthermore, this bi-allelic expression correlated with the increased gene and protein expression of this molecule in women, which can even be detected from peripheral blood mononuclear cells (PBMCs). TLR dosage is considered as a key pathogenic factor in systemic lupus erythematosus (SLE) [38]. Therefore, the increased expression of TLR7 due to XCI escaping might contribute to higher incidences of SLE (90% of SLE patients are women) in women as well as similar skews observed in other auto-immune diseases. Although reports in this area are limited, aging might also affect the XCI patterns and contribute to age-related increases in certain gene expression programs and susceptibility to certain diseases in older women [39]. A future research challenge is to dissect the role of X-linked genes in COVID-19 infections and to understand whether their role is affected with age or with the presence of confounding diseases and conditions (e.g., obesity).

## Sex dimorphism in immune system aging in relation to COVID-19

In addition to aforementioned lifestyle, co-morbidity, and genetic differences, the immune systems of men and women exhibit striking differences especially as individuals age, which might also contribute to the sex dimorphism in COVID-19 cases. Our recent work uncovered how aging affects women’s and men’s immune systems differently [40], in terms of both the extent and the timing of age-related changes. We studied blood-derived immune cells from 172 healthy men and women with ages ranging from 22 to 93, who were carefully screened to exclude confounding diseases, medications, and frailty. Using genomic and functional assays, we uncovered aging-related changes in the number of immune cells, as well as functional differences of these cells by analyzing their transcriptome and epigenome maps. Among aging-related changes shared between sexes, we observed a decline in adaptive immunity (particularly T cells), as well as an activation of innate immunity. Although both men and women exhibited these changes, they were significantly greater in magnitude in men, especially in terms of the activation of innate immunity and inflammation even when the subjects were otherwise healthy and clinically comparable in terms of age, BMI, and ethnicity [40]. In alignment with these genomic findings, serum protein level data from a separate study showed that levels of pro-inflammatory IL6 and IL1RA were higher in older men (65+) compared to older women (*n* = 267 used for re-analyses, 500FG project) [41]. Furthermore, serum levels of these two proteins increased with age more significantly in men compared to women [40]. These data suggest men experience a stronger ‘inflamm-aging’ syndrome than women. Further studies are needed to understand whether increased systemic inflammation in older men could be the foundation of the pro-inflammatory “cytokine storms” that characterize severe overreactions to SARS-CoV-2 virus, a phenomenon more frequently observed in men than women [21]. Based on a recent report from Wuhan China, pregnant women infected with COVID-19 were mostly asymptomatic (92%, 109 out of 118) [3]. Interestingly, the majority of the severe cases among pregnant women (6 out of 9) developed after the delivery, possibly due to the tolerance/anti-inflammatory mechanism pregnant women develop to tolerate the baby during pregnancy [42]. Together these observations suggest that the systemic inflammation levels can be predictive of COVID-19 disease severity. Anti-inflammatory drug treatments (such as anti-IL-6 monoclonal antibodies) are already in clinical trials [24], which might benefit individuals with high levels of systemic inflammation, including older adults – especially older men – and obese individuals (Fig. 1). Efficacy of other anti-inflammatory treatments, particularly the ones that are developed to protect older adults (geroprotective) [43] might be considered in the future for protecting individuals against severe COVID-19 infections.

We also uncovered striking sex differences in how the B cell compartment age [40]. B cells are adaptive immune cells which, upon activation, differentiate into plasma cells and produce antibodies. These antibodies are found in the bloodstream and at the surface of mucosae where they act as an early barrier against infectious agents. Blood B cells (numbers and percentages) were lower in older men (65+) both in our cohort recruited in Connecticut (cell percentages, *n* = 130) and separate cohorts from France (cell percentages and numbers, *n* = 892) [44] and from Japan (cell numbers, *n* = 356) [45], suggesting that some of these sex-differences are conserved across populations. Reduced B cell numbers in older men might result in reduced antibody production that might impair an individual’s ability to fight viruses and other pathogens. In alignment with this, COVID-19 patients with severe symptoms had three times fewer B cells in their blood than asymptomatic patients (109 vs. 373 cells per microliter of blood) [46], similar declining trends were observed in a smaller cohort, albeit not reaching to statistical significance levels (*n* = 44) [21]. In a pilot study of 5 critically ill COVID-19 patients, the administration of plasma from recovered patients that contains antibodies were able to neutralize SARS-CoV-2 virus significantly and improved the patients’ clinical status [47]. Larger antibody-based treatment studies are currently ongoing (Fig. 1). Antibody based treatment strategies may compensate for the B cell deficit of older men. However, a safety concern is a phenomenon called antibody-dependent enhancement, when non-neutralizing antiviral antibodies facilitate entry into host cells thereby increasing the viral infectivity. This phenomenon is observed in vitro and has been found to occur in humans infected with dengue virus or vaccinated with an early Respiratory Syncytial Virus (RSV) vaccine [48]. This has also been observed with anti-spike antibodies causing acute lung injury during acute SARS-CoV infection [49].

We also observed accelerated age-related T cell function declines in men compared to women [40], which was also reported in the Japanese cohort (*n* = 356) [45]. For example, naïve T cell frequencies decreased with age, particularly in CD8^+^ T cells in both sexes, however, women had more naïve T cells compared to men in both young and older subjects, which was also observed in other studies [50]. Women have been shown to have elevated thymic function compared to men at all ages [51], which might potentially explain sex-differences in naïve T cells. Lymphopenia (having reduced numbers of lymphocytes in the blood) has been reported in severe cases of COVID-19 in multiple studies [21, 23, 52, 53], including drastic declines in CD4+ and CD8+ T cells as well as NK and B cells. Together, these data suggest that SARS-CoV-2 might impair antiviral immunity significantly and this impairment might have more severe consequences for older adults. Several anti-viral drugs are in clinical trials for COVID-19, including Remdesivir that was originally developed for Ebola patients; the US Food and Drug Administration (FDA) recently issued an emergency use authorization of Remdesivir for severe COVID-19 cases.

## Timing of age-related changes in the immune system

Finally, by employing statistical algorithms to model temporal trends in genomics data generated from blood samples, we showed that the timing of age-related changes in the immune system differ between men and women. Although we noted a gradual decline in the immune system in both men and women accruing for most of their adult life, these changes accelerated drastically at two time points in life. The first acceleration point occurs in both sexes at around 40 years of age. In the context of COVID-19, this is the age when a doubling in the death rate was reported from 0.2 to 0.4% in China (https://www.worldometers.info/coronavirus/coronavirus-age-sex-demographics/). Our previous work has discovered that this “middle-aged” decline in the immune system occurs in mice also, including a drop in production of adaptive immune cells and bias in hematopoietic stem cell (HSC) differentiation toward over-production of innate immune cells [54], supporting that research using model organisms can help us to understand causative mechanisms for such genomic changes. A second and more dramatic acceleration occurs later in life and exhibits a marked sex difference, being more pronounced and occurring approximately 5 years earlier in men compared to women (early/mid-60s in men versus late-60s in women). Interestingly, this difference between men and women parallels the 5-year gap between sexes in terms of the expected lifespan in the US. The period of accelerated immune changes in the 60s roughly coincides with the uptick in the lethality of COVID-19 reported in China and Italy. These temporal changes reflect declines in adaptive immunity and increases in innate immunity for both sexes. However the magnitude of changes were stronger in men, a finding in alignment with the sex bias in cytokine storms and the mortality of COVID-19 cases. Data from Italy – one of the few countries that report detailed information on sex – also support a gap between sexes in terms of experiencing the lethal effects of COVID-19 infections, where the median age of women who have died from COVID-19 is 82 versus 78 for men (https://www.epicentro.iss.it/coronavirus/). Data and analyses on the timing of immune system changes with age might inform us on when to start interventions and treatments for older adults. Little is currently known as to whether HSC lineage bias diverges between sexes as they age. Understanding the mechanisms by which, and extent to which, HSC lineage bias may be distinct between men and women with aging, and determining if this is a root cause of immune changes between men and women, will be an important area of investigation.

Sex hormones, e.g., testosterone and oestrogen, play diverse roles in immune responses [55], some of which are due to the direct interactions between sex hormones and immune cells. Immune cells can express receptors for sex hormones suggesting that these cells can directly respond to changing hormone levels in our body. For example, estrogen receptors (ERα and ERβ) are expressed in a diverse array of immune cells (T, B, natural killer cells, macrophages, DCs, neutrophils). Furthermore, the effect of sex hormones on immune cell functions are proposed to be dose-dependent [55]. Hence, age or menstrual-cycle dependent changes in the sex hormone levels might affect the interaction between sex hormones and immune cells. Surprisingly, our comparisons of men and women PBMCs at different ages [40] showed that immune profiles between sexes diverge as age increases, even though the hormone levels decrease with age. These findings suggest that sex hormones might play roles in balancing immune responses in men and women and compensate for other inherent differences due to genetics. It is also worth noting that the effect of sex hormones is not independent of the sex chromosomes. For example, the androgen receptor (AR), that is activated by the binding of androgenic hormones (e.g., testosterone) is coded on the X chromosome. Future studies are needed to explore to what extent sex hormones –or lack thereof – contribute to disease severity in COVID-19 or other relevant pathologic conditions (e.g., systemic inflammation).

## Age-related clonal hematopoiesis and COVID-19 co-morbidities

With aging, changes in our hematopoietic (blood) stem cell pool, which can be thought of as the “root” of the immune system, contribute to the functional decline in both our innate and adaptive immune systems. Hematopoietic stem cells (HSCs) accumulate somatic mutations with aging, increasing the chance that one or more HSCs in the pool will accrue a mutation that confers a competitive fitness advantage over other HSCs. This is frequently observed in older individuals, where outgrowth of a mutated HSC and its immune cell progeny is termed ‘clonal hematopoiesis’. In initial reports using exome sequencing to detect clonal hematopoiesis, this condition was detectable in < 1% of individuals under age 40, increasing in frequency in each decade of life up to an incidence of 10–20% of individuals in their 70s or older [56–58]. In addition to the increased prevalence in aging, there are other similarities between populations susceptible to clonal hematopoiesis and COVID-19 morbidity. Clonal hematopoiesis is more commonly observed in older men [57]. Increasing evidence suggests that cardiovascular comorbidities are common in patients with COVID-19, and such patients are at higher risk of morbidity and mortality [59]. The risk of cardiovascular disease more than doubles in individuals with clonal hematopoiesis [57, 60], which is as great or greater than well-described risk factors such low-density lipoprotein (LDL) cholesterol level and blood pressure. Mouse models of common clonal hematopoiesis mutations have established that this link is causative, causing increased size of atherosclerotic lesions and leading to worsening heart function [60–63]. Although large, population-level epidemiological studies directly linking clonal hematopoiesis to COVID-19 outcomes are currently lacking, our understanding of immune system changes caused by clonal hematopoiesis mutations may help to inform the design of studies to determine causality. How might clonal hematopoiesis in aged populations increase their risk of death from COVID-19 infection?

Our work [64] and that from other investigators [64–69] have shown that the most common mutations found in clonal hematopoiesis not only result in a competitive fitness advantage in HSCs but also result in biased differentiation toward innate immune cells at the expense of adaptive immune cells, and propensity for transformation to blood cancers within the innate immune system. Clonal hematopoiesis mutations can increase levels of pro-inflammatory cytokines, including IL-6, IL-1β and IL-8, and inflammatory responses in macrophages and mast cells [60–62, 70, 71]. Elevated levels of pro-inflammatory cytokines, sustained over-production of innate immune cells and reduced production of adaptive immune cells, as a consequence of clonal hematopoiesis, might be contributing to poor COVID-19 outcomes in older adults.

## Systems and predictive immunology to study COVID-19

Systems immunology is an inter-disciplinary field that generates and integrates diverse genomics and phenotypic data to drive a systems-level understanding of immune functions and immune disorders [72]. Recent advances in single cell technologies provide opportunities to study gene and protein expression as well as epigenetic patterns at single cell resolution [73, 74, 80]. Systems-level studies some of which by taking advantage of these single cell technologies have already been impactful in diverse immune-related conditions including but not limited to age-related decline in immune functions [5, 14, 40, 75] and vaccine responsiveness [76], auto-immune diseases, [77, 78], and cancer [79, 80]. Generation of such datasets from human immune cells [81] in the context of COVID-19 and proper integration of these data using advanced computational techniques [73] will be instrumental in answering some of the open questions related to the current health crisis. Particularly, systems-level approaches can be effective in i) assessing risk at the individual level and dissecting how different factors contribute to this risk (age, sex, BMI); and ii) understanding genomic or cellular predictors of responsiveness to potential therapies. Furthermore, integrating these single cell genomic technologies with genotypes of individuals can bring further resolution related to the role genetics play in disease risk and in selecting appropriate therapies tailored for individual patients [82, 83].

## Conclusions

Emerging data collected from COVID-19 patients together with our findings from healthy donors drive home a critical point: an improved understanding of sex-specific and age-dependent immune characteristics will lead to more informed and ultimately more effective clinical decisions. Profiling an individual’s blood-derived immune cells is easily accessible and a relatively cheap way to assess health and disease risk at the individual level. Recent advances in genomics have led to powerful tools to study the human immune system down to the single cell level resolution. What we can learn from a small amount of blood sample when combined with these technologies is unprecedented in detail. Furthermore, advances in computing methods (e.g., deep learning algorithms), provide us with a unique opportunity to analyze and interpret these data to assess health and disease risk in an increasingly personalized and precise manner. While we currently find ourselves in a moment of uncertainty and desperation, we rest certain that the future of medicine is bright given these technological advances that can be harnessed to better understand and cure diseases, including COVID-19 that set a new research agenda for many scientists [84].

## Data Availability

Not applicable.
